# Tuberculin

**Published:** 1899-06-03

**Authors:** 


### TUBERCULIN.

Perhaps one of the most interesting points in the discussion on treatment at the Tuberculosis Congress was the attempt to rehabilitate tuberculin. Dr. Brieger, speaking on this subject, showed that the pessimistic reaction against specific treatment which had followed the enthusiasm created by Koch's first discoveries was absolutely unjustifiable, and he maintained that tuberculin had a strong healing power if the course of treatment were persisted in long enough, even in cases where secondary infection had already set in.

